# Functional Organization and Adaptability of a Decision-Making Network in *Aplysia*

**DOI:** 10.3389/fnins.2012.00113

**Published:** 2012-07-26

**Authors:** Romuald Nargeot, John Simmers

**Affiliations:** ^1^Institut de Neurosciences Cognitives et Intégratives d’Aquitaine, Université Bordeaux, UMR 5287Bordeaux, France; ^2^CNRS, Institut de Neurosciences Cognitives et Intégratives d’Aquitaine, UMR 5287Bordeaux, France

**Keywords:** *Aplysia*, feeding behavior, occasion setting, motor pattern selection, learning, central pattern generator, oscillatory properties, plasticity

## Abstract

Whereas major insights into the neuronal basis of adaptive behavior have been gained from the study of automatic behaviors, including reflexive and rhythmic motor acts, the neural substrates for goal-directed behaviors in which decision-making about action selection and initiation are crucial, remain poorly understood. However, the mollusk *Aplysia* is proving to be increasingly relevant to redressing this issue. The functional properties of the central circuits that govern this animal’s goal-directed feeding behavior and particularly the neural processes underlying the selection and initiation of specific feeding actions are becoming understood. In addition to relying on the intrinsic operation of central networks, goal-directed behaviors depend on external sensory inputs that through associative learning are able to shape decision-making strategies. Here, we will review recent findings on the functional design of the central network that generates *Aplysia’s* feeding-related movements and the sensory-derived plasticity that through learning can modify the selection and initiation of appropriate action. The animal’s feeding behavior and the implications of decision-making will be briefly described. The functional design of the underlying buccal network will then be used to illustrate how cellular diversity and the coordination of neuronal burst activity provide substrates for decision-making. The contribution of specific synaptic and neuronal membrane properties within the buccal circuit will also be discussed in terms of their role in motor pattern selection and initiation. The ability of learning to “rigidify” these synaptic and cellular properties so as to regularize network operation and lead to the expression of stereotyped rhythmic behavior will then be described. Finally, these aspects will be drawn into a conceptual framework of how *Aplysia’s* goal-directed circuitry compares to the central pattern generating networks for invertebrate rhythmic behaviors.

## Introduction

In a relatively constant environment, animals can express variable motor actions as a consequence of internal drives arising from the dynamic properties of central networks. These adaptive behaviors can be rhythmic and relatively stereotyped, or may be highly variable and directed toward a specific goal (Dickinson and Balleine, 1994; Marder, 2000; Pearson, 2000; Brembs, 2011a). Feeding, sexual, and aggressive behaviors in both invertebrates and vertebrates typify such goal-directed actions in which internally derived decisions to act are crucial for the spontaneous expression of behaviorally relevant action patterns (Kupfermann, 1974; Edwards et al., 2003; Dickson, 2008; von Philipsborn et al., 2011). The decision to act implies that the underlying central network possesses the structural and functional mechanisms that autonomously enable the selection of a particular action pattern from several variants and the “setting of the occasion” for its expression (Schall, 2005). Nevertheless, the central network operations that define decision-making are subject to regulation by sensory inputs and the positive (rewarding) or negative (punishing) consequences of an executed action. Through sensory feedback and associative learning, past experience modifies the internal drives which select and set the occasion for action pattern production (Baxter and Byrne, 2006; Brembs, 2011b; Kennerley and Walton, 2011; Nargeot and Simmers, 2011). Although several studies have begun to analyze the neuronal circuits implicated in such behavioral decision-making (Kristan, 2008; Gaudry and Kristan, 2009; Kemenes, 2009; Balleine and O’Doherty, 2010), it remains unclear how these circuits are able spontaneously to generate and organize the neuronal activity underlying coherent occasion setting and action pattern selection, and how these decision-making processes are regulated by learning.

Most of our current knowledge on the ability of the central nervous system (CNS) to spontaneously generate patterned motor activity has derived from the analysis of rhythmic and essentially stereotyped behaviors, such as locomotion and respiration. From these studies, a number of rhythmogenic networks, so-called “central pattern generators” (CPGs), have been identified and the synaptic and intrinsic membrane properties of their constituent neurons defined (for reviews, see Calabrese, 1995; Marder and Bucher, 2001, 2007; Nusbaum and Beenhakker, 2002; Marder et al., 2005; Harris-Warrick, 2010). However, although the ongoing operation of such automatic CPGs can be dynamically regulated by sensory and modulatory inputs, this functional variability is not determined by mechanisms associated with any form of decision-making (Pearson, 2000; Harris-Warrick, 2011).

Insights into the functional design and properties of motor networks that are able autonomously to elaborate action pattern selection and occasion setting in their expression are beginning to emerge for the circuits governing invertebrate feeding behavior. Specifically, an increasing amount of data has allowed characterizing the synaptic organization, cellular properties, and dynamic operation of such networks in mollusks (Elliott and Susswein, 2002; Kemenes and Benjamin, 2009; Nargeot and Simmers, 2011). Here, we will review recent findings on the neuronal constructs of the buccal network which contribute to the autonomous genesis and selection of distinct feeding-related action patterns in *Aplysia*. Evidence that this goal-directed behavior, and the variable motor strategies used in the effective search for food, depends partly on internal and autonomous mechanisms will be presented. Then, the structural and functional properties of the networks mediating this motor variability will be described in order to pinpoint common and distinguishing features with previously characterized CPG networks for automatic behaviors. Particular emphasis will be placed on the contribution of neuronal diversity, and the erratic membrane properties and intercellular connections of identified neurons that select and set the occasions for motor pattern genesis. Finally, the regulation of these fundamental parameters of decision-making buccal circuitry by associative learning will be described.

## Spontaneous Variability in *Aplysia’s* Feeding Behavior

**Figure 1:**
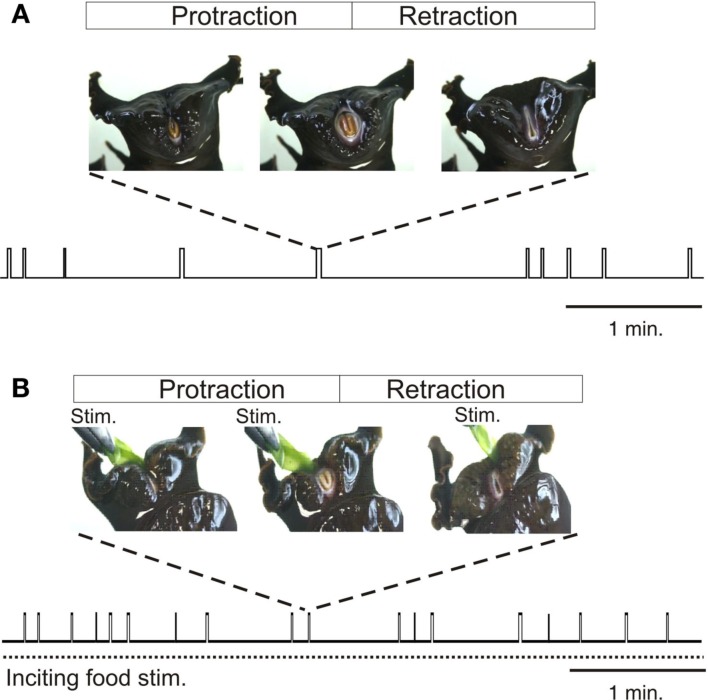
***Radula* movements in freely behaving *Aplysia***. In the absence of food **(A)** or under continuous inciting stimulation with non-ingested food applied to the lips [Stim., **(B)**], *Aplysia* spontaneously generates repeated cycles of protraction and retraction of its tongue-like radula (vertical bars). The distribution in time and the duration of radula movement cycles are highly variable and are not determined by any explicit sensory cue, although the mean frequency of cycle occurrences increases during inciting stimulation. Between successive radula cycles the animal engages in various other actions including lip movements, head-waving or locomotion which are all directed toward effective food seeking.

In searching for food, the herbivorous *Aplysia* performs a variety of motor acts including locomotion, postural movement, head-waving, and buccal movements, which although variable in terms of occurrence, duration, and intensity, are all directed toward the goal of obtaining appropriate nutriment (Kupfermann, 1974). Past studies have focused on buccal movements, and particularly those of the tongue-like radula, as they are easily observable and quantifiable during ongoing feeding behavior (Figure 1). *Radula* movements are organized in repeating cycles that each consists of three successive actions: a protraction phase, retraction phase, and closure of the appendage’s two halves (Morton and Chiel, 1993a; Neustadter et al., 2002; Horn et al., 2004). Depending on the relative durations of these phases and the timing of closure activity, a radula movement cycle can engage in at least two distinct behaviors – ingestion (biting, swallowing) and egestion. In an ingestive cycle, following a short protraction, radula closure that serves to grasp food, occurs mainly during a prolonged retraction phase, thereby drawing particles into the buccal cavity. In an egestive cycle, radula closure now occurs mainly during the extended protraction phase that precedes a shorter retraction, thus withdrawing particles back out of the buccal cavity. These different radula actions are expressed spontaneously in the absence of any food stimulus when the animal is randomly sampling its environment (Figure 1A), or they occur at an elevated mean frequency in the continuous presence of a non-ingestible food stimulus (Figure 1B).

In freely behaving animals, both the expression and temporal features of radula movement cycles are highly variable within a given feeding sequence. This behavioral variability is evident in the changing delay with which a transient food stimulus triggers a given radula cycle (Susswein et al., 1976), and in the unpredictable occurrence and structure of cycle emissions in the absence or presence of a constant food stimulus (Horn et al., 2004; Lum et al., 2005; Brezina et al., 2006; Nargeot et al., 2007). Thus, successive radula movements occur in an unpredictable mixture of opposing ingestive and egestive action patterns, and the intervals between the onsets of successive cycles may vary considerably, without being related to any explicit sensory cue (Figure 1). In addition to this spontaneous variability in the initiation and selection of radula actions, the movements within individual cycles (protraction, retraction, closure) change considerably on a cycle-to-cycle basis in terms of their duration and strength. This flexibility does not correspond to random noise in feeding behavior, but rather, is associated with the changing efficiency with which the radula is cyclically protracted and retracted in a trial-error strategy directed toward the successful seeking and consumption of food (Lum et al., 2005).

Without excluding the possible contribution of extrinsic sensory information in this behavioral flexibility via influences on both the initiation and selection of radula action, several lines of evidence suggest that the motor variability arises primarily from an autonomous central process for accomplishing effective feeding. First, the variability in feeding behavior occurs *in vivo* in the absence of any food stimulus (Nargeot et al., 2007). Second, the parameters that characterize the behavioral irregularity continue to be expressed by radula output patterns in isolated neuromuscular and *in vitro* CNS preparations (Morton and Chiel, 1993b; Nargeot et al., 1997; Horn et al., 2004; Zhurov et al., 2005). Third, in the isolated buccal nervous system, essential aspects of the behavioral variability were found to be correlated to spontaneous, cycle-to-cycle changes in the bioelectrical activity of identified elements of the underlying central network (Hurwitz et al., 1997;Nargeot et al., 1999a,b, 2009; Jing and Weiss, 2005). Fourth, learning processes which substantially diminish or even suppress the behavioral irregularity induce corresponding changes in the endogenous properties of the network’s neurons (see below). Thus, a major determinant of the spontaneous irregularity in *Aplysia’s* feeding behavior appear to be encoded within the functional properties of the elements comprising the central circuitry that autonomously organizes, selects, and sets the occasion for the production of goal-directed movement cycles.

## Organizational Properties of a Multifunctional Network

**Figure 2:**
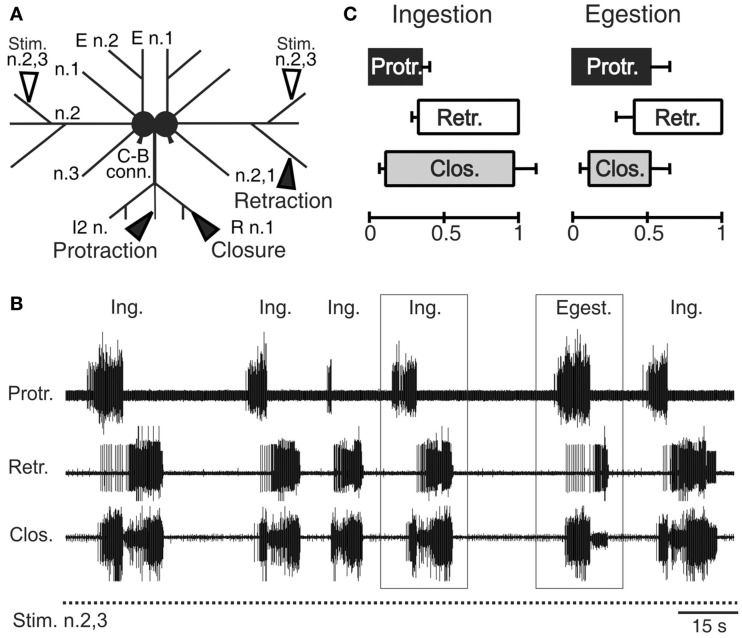
***Radula* motor pattern generation in isolated buccal ganglia**. **(A)** Schematic of the *in vitro* ganglia. Stimulating electrodes (unfilled arrowheads) are positioned on the buccal nerves 2,3 (n.2,3) containing sensory fibers that are normally activated by food stimuli within the anterior part of the buccal cavity (Nargeot et al., 1997). Recording electrodes (filled arrow heads) are positioned on the intrinsic nerve 2 (I2 n.), the buccal nerve 2,1 (n.2,1) and the radula nerve (R n.), which carry axons of protractor, retractor, and closure motor neurons, respectively. **(B)** Simultaneous extracellular recordings of spontaneous cycles (two are indicated by rectangles) of radula protractor (Protr.), retractor (Retr.), and closure (Clos.) motor activity during tonic (2 Hz) inciting stimulation of the input nerve 2,3 (Stim. n.2,3). The distribution in time of these radula motor pattern cycles and the durations of the different activity phases are highly variable [see also **(C)**] and are not determined by any sensory cue. **(C)** Based on the relative durations of the protraction/retraction phases and the phase position of closure activity, two distinct motor patterns are distinguishable (also see rectangles in **(B)**] that correspond to ingestion (Ing.) and egestion (Egest.) radula movements during actual feeding in the intact animal.

The interneurons, motor, and sensory neurons responsible for producing and adjusting radula movements are distributed in two interconnected and essentially identical neuronal circuits located within the bilateral buccal ganglia situated on the buccal mass (Kupfermann, 1974; Elliott and Susswein, 2002). In isolated *in vitro* buccal ganglia preparations, whether spontaneously active or subjected to tonic electrical stimulation of peripheral sensory nerves, this bilateral network continues to generate the motor patterns that underlie the essential features of radula movements and their variability observed in the freely behaving animal (Figures 2A,B; Morton and Chiel, 1993b; Nargeot et al., 1997; Jing et al., 2011). These “fictive” *in vitro* patterns are therefore composed of successive protraction and retraction phases of changeable durations and in variable overlap with closure motor nerve discharge. Thus, according to the phase position of the latter and the relative durations of the protraction and retraction phases, the distinct motor patterns that normally produce ingestive or egestive movements can be readily distinguished (Figures 2B,C).

In correspondence with actual behavior, the selection, initiation, and structure of radula motor patterns recorded from *in vitro* preparations also vary spontaneously from cycle-to-cycle, and in a highly irregular manner (Figure 2B). Not only are successive patterns comprised of burst activities of variable durations and frequencies, but the pattern phenotype (i.e., fictive ingestion or egestion) and the interval between the onsets of successive patterns varies considerably and unpredictably. Thus, in isolation, the multifunctional buccal network is able to autonomously organize, select, and set the occasions for emitting the distinct biologically relevant patterns of radula motor activity that occur *in vivo*.

### Basic constructs of the buccal network

**Figure 3:**
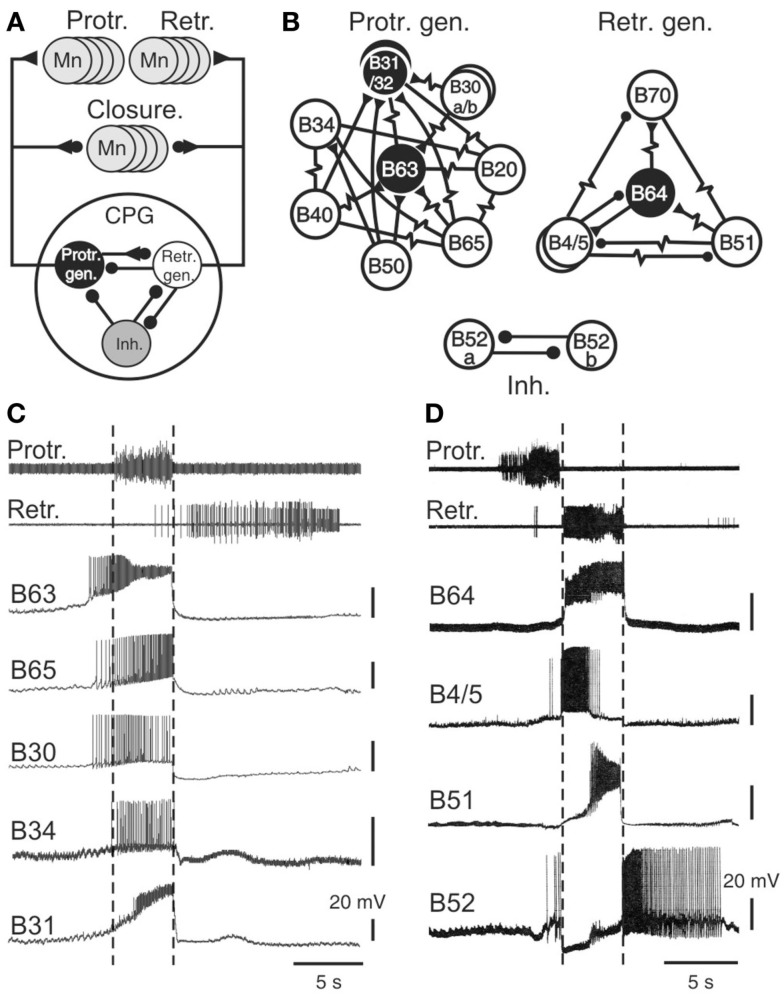
**The radula motor pattern generating network**. **(A)** Simplified schematic of the buccal central pattern generator (CPG) and its synaptic connections with protractor, retractor, and closure motor neurons (Mn); the small filled circles represent inhibitory connections while triangles denote excitatory connections. The CPG is composed of three distinct and interconnected groups of neurons: a protractor generator (Prot. gen.), retractor generator (Retr. gen.), and a group of inhibitory neurons (Inh.). **(B)** Detailed schematics showing the neuronal diversity and synaptic connectivity within the protractor and retractor generators, and the inhibitory group. Neurons that are necessary and sufficient for radula motor pattern genesis are in black (see text). Simple resistor symbols represent non-rectifying electrical coupling; resistor symbols associated with a circle or triangle represent non-rectifying electrical coupling associated with an excitatory or inhibitory chemical synapse, respectively. For simplification, not all known synaptic connections including those between the different neuronal groups of neurons [see **(A)**] are shown. **(C)** Simultaneous extracellular recordings of radula protraction and retraction motor output and intracellular recordings from neurons of the protractor generator. The B34 and B31 cells were recorded in a different preparation from the other protractor neurons, but during a motor pattern of similar protractor phase duration. Note that the burst onsets of B63, B65, and B30 anticipate protractor motor nerve activity (indicated by vertical dotted lines). **(D)** Simultaneous extracellular recordings of protraction/retraction motor output and intracellular recordings of retractor generator neurons (B64, B4, B51) and an inhibitory neuron (B52).

The key components of the buccal feeding network have been identified and characterized in a variety of cellular studies using intracellular recordings (Gardner, 1977; Susswein and Byrne, 1988; Plummer and Kirk, 1990; Hurwitz and Susswein, 1996; Hurwitz et al., 1997; Kabotyanski et al., 1998; Jing and Weiss, 2002; Dembrow et al., 2004; Jing et al., 2004; Sasaki et al., 2009; see also Elliott and Susswein, 2002). Together these neurons constitute a pattern generating ensemble that shares several features with previously described CPGs for automatic rhythmic behaviors in invertebrates (Figure 3A; Getting, 1989; Nusbaum and Beenhakker, 2002; Marder and Bucher, 2007; Selverston, 2010). First, buccal circuit neurons generate spontaneous bursts of action potentials that are associated with at least one of the phases of overall network output. Second, experimental manipulation of their electrical activity can modify the cycle frequency of motor pattern expression via influences on two fundamental network properties: the intrinsic bioelectrical properties of constituent neurons and their synaptic interconnections (Figures 3B,C). As in other CPGs, specific membrane properties of buccal CPG neurons include post-inhibitory rebound, regenerative plateauing mechanisms, and endogenous oscillatory properties (Plummer and Kirk, 1990; Evans et al., 1999; Susswein et al., 2002; Nargeot et al., 2009) that underlie burst generation and autonomous network functioning. In addition, the coordination of cellular bursting into behaviorally appropriate motor output is conferred by the synaptic connections among the circuit neurons. Network synapses are generally reciprocal, although individual neurons may also exert complex synaptic influences on variable proportions of their circuit partners via a diversity of electrical and/or chemical, excitatory and/or inhibitory, conventional fast and/or modulatory actions.

Sixteen bilateral pairs of identified cells, including sensory, interneurons, and motor neurons, have been classified as integral members of the buccal CPG (Figures 3A,B). They are grouped into three different functional subsets, each dedicated to the genesis of a specific phase of buccal motor activity. The protractor generator subset contains those neurons that are active during, and trigger aspects of protractor motor output (Susswein and Byrne, 1988; Hurwitz et al., 1997; Kabotyanski et al., 1998; Jing and Weiss, 2002; Dembrow et al., 2004; Jing et al., 2004), while the retractor generator contains the corresponding neurons for retractor output (Gardner, 1977; Plummer and Kirk, 1990; Hurwitz and Susswein, 1996; Sasaki et al., 2009). These two functional groups are connected by reciprocal synapses that ensure the strict succession of protraction and retraction phases of activity. A third neuronal subset, composed of inhibitory cells, transiently inhibits both the protractor and retractor generators and thereby terminates each pattern cycle (Plummer and Kirk, 1990; Evans et al., 1999; Nargeot et al., 2002). Most of these CPG neurons are monosynaptically connected to corresponding populations of motor neurons controlling radula protraction and retraction (Church and Lloyd, 1994; see Elliott and Susswein, 2002), while several CPG elements excite motor neurons that drive closure muscle contractions in phase with protraction and/or retraction movement.

### Functional diversity of buccal network neurons

A striking feature of buccal network design is the diversity of neuron types within each functional subset (Figures 3B–D). For example, of the 10 bilateral pairs of protractor generator neurons, 8 of these are distinctly different in terms of their intrinsic membrane properties, patterns of synaptic connectivity, and specific roles in the generation of protractor and closure motor activity. Similarly, four of the five bilateral cell pairs in the retractor generator differ significantly in their membrane properties, synaptic connections and contributions to retractor, and closure output. Although the neurons comprising each functional group are most often coupled via electrical or excitatory chemical synapses, due to a general weakness of these connections and/or individual differences in membrane properties, cells within a given group are able to express impulse bursting with distinct durations, frequency, and timing (Figures 3C,D).

In addition to a functional division according to their participation in the different phases of radula motor pattern production, buccal CPG neurons also play primary or secondary roles in the actual pattern generating process, with the former being necessary and sufficient for producing a specific pattern phase while the latter are not. Essential (primary) roles are restricted to the B63 and B31/32 neurons of the protractor generator and the B64 cell of the retractor generator (Figure 3B), their spontaneous impulse bursts being responsible for triggering the protraction and retraction phases, respectively. Accordingly, an experimental hyperpolarization by intracellular current injection of any one of these cells to prevent its bursting activity suppresses production of the corresponding phase of buccal output. Conversely, an experimental depolarization of a previously silent essential cell triggers its bursting and instigates the corresponding phase of radula activity.

The secondary cell subtype contributes to motor pattern genesis but is not necessary for its expression. This group includes the B20, B30, B34, B40, B50, B65 neurons of the protractor generator, and the B4/5, B51, B70 cells of the retractor generator. Similar to CPG neurons generally, these non-essential elements can produce spontaneous bursting in time with buccal motor output and their experimental depolarization in an otherwise silent preparation can trigger protraction or retraction phases of activity. However, in contrast to the essential B63, B31/32, and B64 neurons, a hyperpolarization to prevent their spontaneous bursting does not impair overall motor pattern genesis. Moreover, about half of these follower neurons (specifically B30, B34, B40, B50, B51) are not systemically active during successive cycles of radula motor output, further indicating that these cells serve as occasional contributors to setting the intensity and/or type of pattern expressed (see for example, B51 in Figure 5A).

Although *Aplysia’s* feeding network shares common organizational and functional features with CPG networks responsible for more stereotyped rhythmic behaviors, the latters’ functional subcircuits are usually considered to be composed of individual neurons or cell assemblies that are similarly necessary and sufficient for motor pattern genesis (Getting, 1989; Syed et al., 1990; Marder and Calabrese, 1996). Furthermore, the cellular components of a given functional group are most often strongly connected through electrical coupling or excitatory chemical synapses, thereby ensuring tightly coordinated, and often synchronized, bioelectrical activity throughout the subset. In the buccal CPG, by contrast, necessity, and sufficiency for generating the protraction and retraction phases of radula motor output is vested in small neuronal subpopulations of the wider feeding network. This essential kernel is synaptically connected to the remaining cohort of second-order neurons that also have specific synaptic connections, membrane properties and patterns of firing, and which thereby exert varying influences on the activity of the essential neurons and motor pattern genesis. Thus, an important distinguishing feature between the multifunctional network responsible for *Aplysia’s* goal-directed feeding behavior and CPGs engaged in stereotyped automatic behaviors is the neuronal diversity that, by imparting cycle-to-cycle variability to the motor command, provides the substrate for a potential decision-making capability for when (occasion setting) and how to act (motor selection).

## Occasion Setting Via the Association of Asynchronous Bursting and Irregular Membrane Properties

The buccal CPG elements that set the occasions for radula motor pattern expression would be reasonably expected to be those cells that are active before or during protraction activity, the initial phase of each pattern cycle. Within the protractor generator circuit, three neuron pairs have been found to generate spontaneous impulse bursts with onsets that consistently precede the protraction phase of each buccal motor pattern by up to several seconds (Nargeot et al., 2009). This anticipatory cell group consists of the essential B63 neurons and the optional neurons B30 and B65 (Figure 3C). The B63 cells are electrically coupled to the latter (Kabotyanski et al., 1998; Jing et al., 2004; Nargeot et al., 2009) and all three cell types make excitatory synapses with protractor motor neurons B31/32. However, due to their particular membrane properties and monosynaptic connectivity with other CPG elements and motor neurons, bursting in each of these anticipatory neurons contributes differently to buccal network operation (Nargeot et al., 2009). For example, spike bursts induced by depolarizing current injection into a previously silent B63 neuron are able to elicit complete motor patterns, without necessarily triggering activity in B30 and B65. In contrast, burst discharge evoked in either B30 or B65 systematically triggers bursting in B63 and consequently motor pattern emission. Alternatively, when B63 is held silent with hyperpolarizing current, an experimental activation of either B30 or B65 is no longer able to elicit a complete pattern, and at most, only closure or protractor motor output, respectively, occurs. Thus, the anticipatory protractor neurons constitute a heterogeneous subset of electrically coupled neurons of which the B63 cell pair, activated intrinsically or driven extrinsically by the B30 or B65 neurons, are the sole necessary instigators for motor pattern production.

**Figure 4:**
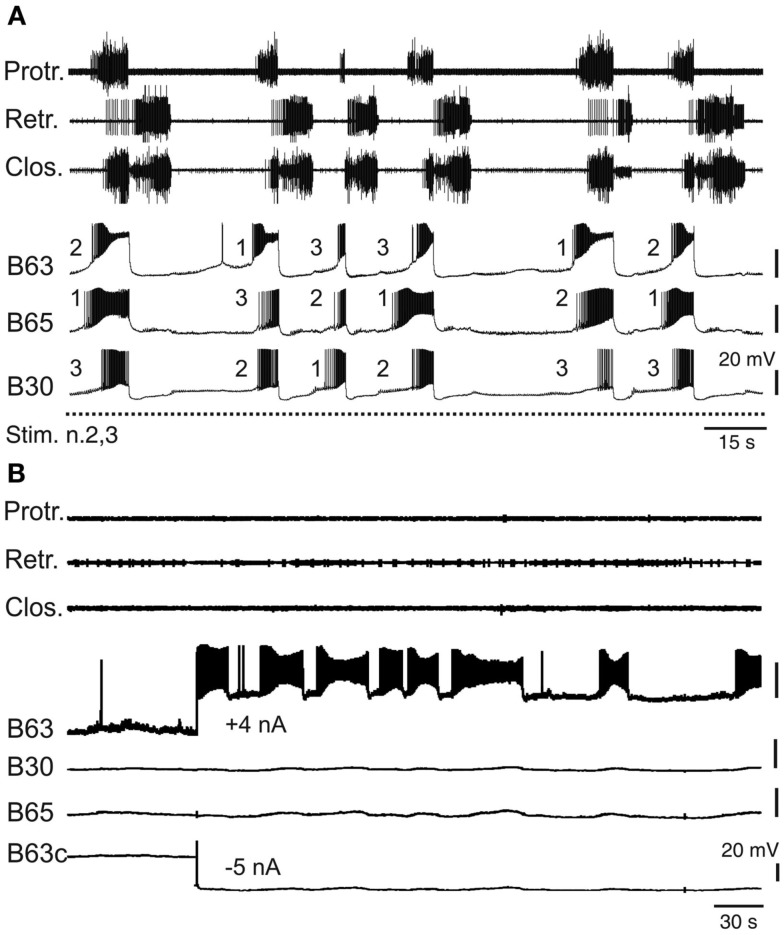
**Variability of endogenous anticipatory neuron bursting**. **(A)** Simultaneous extracellular recordings of radula motor patterns (top three traces, same layout as in Figure 2B) and intracellular recordings of protraction initiating neurons (B63, B65, B30) during tonic (2 Hz) stimulation of n.2,3 (Stim. n.2,3). The irregular occurrences of motor patterns were associated with uncoordinated bursting and cycle-to-cycle variability in the order of burst onsets (indicated by numbering) in B63, B65, B30 prior to each motor pattern emission. **(B)** Endogenous bursting capability of an anticipatory B63 neuron under conditions of functional isolation *in situ*. In the absence of n.2,3 stimulation and with the contralateral B63 (B63c) held hyperpolarized (by −5 nA current injection) to prevent buccal CPG operation and therefore motor pattern emissions (see top three traces), a tonic depolarization of the other B63 (with +4 nA injected current) elicited repetitive, albeit erratic, bursting in the anticipatory cell.

Despite the electrical coupling between these anticipatory neurons, the onsets of their spontaneous bursts are generally highly uncoordinated and vary randomly on a cycle-to-cycle basis (Nargeot et al., 2009). Consequently, the first active cell as well as the order in which bursting commences in the other subset partners can change considerably and in an unpredictable manner from one cycle to another (Figure 4A). Several lines of evidence indicate that this lack of coordination is the major determinant of the variability in the central drive that sets the occasions for radula motor pattern emissions. First, a regularization of spontaneous radula movement cycles *in vivo* and of the underlying motor patterns *in vitro* can be induced by operant learning processes (see below). This stabilization of buccal CPG output was found to be correlated with an increased coordination of burst onsets in the B63, B30, B65 neurons, whereby bursts in the essential neurons B63 now invariably precede, with a brief delay, the burst activity of the other anticipatory cells in each motor pattern cycle (Nargeot et al., 2009). Moreover, this learning-induced cellular plasticity is associated with an increase in the electrical coupling amongst the anticipatory neuron subset. Second, and in a related manner, the highly erratic genesis of buccal motor patterns can be switched to regular rhythmic emissions by experimentally enhancing the electrical coupling between the anticipatory neurons: in isolated buccal ganglia preparations, an artificial increase in the electrical coupling of B63-B30 and B63-B65 was found to coordinate their bursting activity and regularize the subsequent expression of radula pattern cycles (Nargeot et al., 2010; Sieling et al., 2010; also see below).

Bursting in the anticipatory neurons, which occurs in absence of sensory input or under tonic sensory nerve stimulation, is driven principally by an endogenous oscillatory mechanism that was previously revealed under conditions of *in situ* isolation of individual cells (Figure 4B) by continuously hyperpolarizing a single B63 neuron to block activity in the protractor generator and the remaining CPG network (Nargeot et al., 2009). In such functional isolation, each anticipatory neuron was found capable of generating repetitive bursts of action potentials when subjected to tonic depolarizing current injection. Moreover, in close accordance with the normal erratic emissions of radula motor patterns, bursting in isolated neurons occurred with highly irregular durations and inter-burst intervals (Figure 4B). Thus, this erratically expressed intrinsic property of the anticipatory neurons, in combination with their weak electrical coupling, offer plausible substrates for the irregular central drive that sets the occasions for the expression of radula movements *in vivo*. Interestingly, the decision to act has also been partly attributed to the B31/32 neurons that are synaptically coupled to the B63, B30, B65 subset and which govern the transition between the onset of anticipatory activity and protractor phase production. The relative timing of this transition is highly variable and again is dependent on the active contribution of membrane conductances that in this case are specific to the B31/32 neurons (Hurwitz et al., 2008).

Together these findings therefore indicate that the irregular bioelectric behavior of a heterogeneous and asynchronously active core circuit can provide the internal drive that autonomously sets the unpredictable occasions to act in a goal-directed behavior.

## Motor Pattern Selection by Central Network Reconfiguration

In a given feeding sequence, *Aplysia* expresses different and even opposing action patterns that underlie cyclic ingestive and egestive radula movements. The animal’ ability to switch between these two behaviors is presumably related to its trial-and-error feeding strategy, serving as a maneuver to more efficiently shear off food particles or to ensure the radula’s correct alignment in the buccal cavity (Horn et al., 2004; Lum et al., 2005). Again, while the “choice” of radula action must be adaptable to the sensory environment, several arguments indicate that the selection process is mainly conferred by the inherent functional properties of the buccal CPG network itself. In the absence of sensory stimulation or under a constant electrical activation of peripheral input nerves, the isolated buccal ganglia continue autonomously and interchangeably to emit the motor patterns that underlie ingestive and egestive movements *in vivo* (Nargeot et al., 1997; Horn et al., 2004). Thus, buccal circuitry not only inherently sets the occasion for motor pattern emission, but also decides on the specific pattern phenotype expressed.

**Figure 5:**
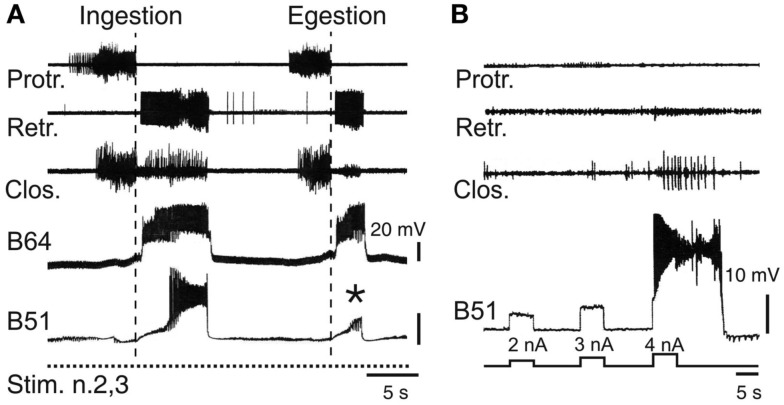
**Neuronal correlates of spontaneous motor pattern switching**. **(A)** Extracellular recordings of radula motor patterns (top three traces) and intracellular recordings of the essential B64 neuron and the optional B51 cell of the retractor generator. Switching between ingestion and egestion motor patterns during tonic (2 Hz) stimulation of n.2,3 (Stim. n.2,3) was associated with the spontaneous, all-or-none activation or absence (*) of bursting in B51. **(B)** Endogenous plateau property of B51 revealed by brief intracellular current injections in the absence of n.2,3 stimulation. Note that a burst-generating plateau in B51 also elicited closure motor activity that normally occurs during the retraction phase of ingestion patterns [see **(A)**].

The clearly distinguishable features of ingestive and egestive outputs have enabled the neuronal basis of this pattern selection process to be investigated *in vitro* (Figure 5A). Intracellular recordings have identified several neurons within the buccal network that are responsible for specifying ingestion versus egestion motor patterns (Hurwitz et al., 1997; Kabotyanski et al., 1998;Nargeot et al., 1999a,b, 2002; Cropper et al., 2004; Jing et al., 2004). In contrast to the anticipatory cell kernel, which participates in all buccal motor patterns and thereby contributes to their common features such as protraction and retraction phase alternation, the CPG neurons involved in pattern selection are able to regulate the durations of the protraction and retraction phases and their temporal relationship with radula closure activity. These latter cells, which are not essential for motor pattern genesis, are only active during the expression of a specific pattern. A well-established example is the bilateral pair of B51 neurons (Nargeot et al., 1999a), which remain inactive during egestion pattern genesis, but fire intense bursts during ingestion patterns (Figure 5A). This pattern-specific bursting of B51 in turn triggers closure motor activity in phase with the prolonged retraction phase of the ingestion pattern via monosynaptic excitation of the B8 closure motor neurons and the essential B64 retractor generator neuron (Nargeot et al., 1999b). Significantly, a brief sub-threshold depolarization of B51 by current injection during each spontaneous pattern emission was found to bias buccal circuit output toward expression of the ingestion pattern. Conversely, suppression of B51 activity with transient hyperpolarizing current biases the selection process toward non-ingestive pattern emissions. Similarly, other neurons of the buccal network (B34, B52) only generate bursts during the protraction phase of egestion patterns and through their synaptic connections with closure motor neurons and protractor or retractor generator neurons, are able to instruct the buccal CPG to produce egestive behavior (Hurwitz et al., 1997; Nargeot et al., 2002).

Thus, motor pattern selection in the multifunctional buccal network is determined, at least in part, by the dynamic recruitment of specific components whose activity defines the different phenotypes of circuit output. On this basis, the buccal CPG does not constitute a prescribed and constant population of reliably active neurons responsible for generating a single program of motor output. Rather, the functional composition of the network varies substantially on a cycle-to-cycle basis and in an unpredictable manner, thereby specifying individual motor programs from the variants that the overall circuit is capable of producing. It is well known that CPGs in general are not fixed entities, but that depending on sensory or modulatory influences, individual neurons can be dynamically recruited into, or excluded from, a given network in order to generate different forms of the same behavior, or even distinct behaviors (Hooper and Moulins, 1989; Dickinson et al., 1990; Meyrand et al., 1991; Nargeot, 2001; see also Morton and Chiel, 1994; Cropper et al., 2004; Harris-Warrick, 2011). However, in contrast to such extrinsically instructed circuit reconfigurations, the buccal CPG is capable of a remodeling that can occur independently of external sensory or modulatory cues, but instead it arises from the particular synaptic and active membrane properties of the circuit neurons themselves. In the case of the B51 neuron, for example, its pattern-selecting burst occurrences derive from the cell’s electrical coupling with the retractor generator B64 neuron, which in turn triggers all-or-none firing in B51 as a result of the latter’s intrinsic plateauing property (Figure 5B; Nargeot et al., 1999b).

## Learning-Induced Rigidification of Buccal Network Functioning

Although the motor patterns for motivated behaviors can originate autonomously from the underlying central networks, the internally driven incentive to act is regulated by sensory inputs and learning. Associative learning, including both classical and operant conditioning, plays a critical role in altering the neuronal processes that set and select behavioral action (Taylor and Lukowiak, 2000; Brembs, 2003, 2011b; Baxter and Byrne, 2006; Balleine and O’Doherty, 2010; Nargeot and Simmers, 2011).

In appetitive classical conditioning of *Aplysia’s* feeding behavior, pairing an unconditional food stimulus with a tactile conditional stimulus (CS) to the lips increases the probability of a subsequent CS to elicit an ingestive radula cycle (Lechner et al., 2000a). The basis for this learning is a synaptic facilitation which enhanced ability of the CS pathway to trigger the motor pattern-initiating neurons B31/32 and pattern-selecting bursts in the B51 neuron (Lechner et al., 2000b; Mozzachiodi et al., 2003; Lorenzetti et al., 2006). Thus, through a pairing-specific occasion setting for a feeding response via B31/32, and pattern selection by B51, the buccal CPG is “instructed” to more reliably produce output of an ingestive nature.

Learning not only modifies sensory-elicited responses, but may also regulate the internally driven impulse for motor pattern production. In operant conditioning, an animal learns to make the contingent association between the spontaneous emissions of an action and its outcome (either rewarding or punishing). As a consequence, the probability of the designated behavior’s expression is persistently modified and in some cases, particularly with highly appetitive rewards, may lead to a rhythmic, compulsive-like expression of the rewarded action.

Several operant conditioning paradigms have also been developed for *Aplysia’s* feeding behavior and the resulting plasticity analyzed at the cellular and network levels (Susswein et al., 1986; Brembs et al., 2002; Nargeot et al., 2007). In an appetitive form of this learning, spontaneous ingestive radula cycles were associated with the delivery of a food reward during training. After several minutes of such action/reward associations, the rate of the spontaneous occurrences of ingestive cycles was found to increase dramatically and the highly irregular expression of ingestive motor patterns switched for several hours to regular rhythmic occurrences (Nargeot et al., 2007). This behavioral plasticity was not observed when the food reward was replaced by a neutral food stimulus or when the reward was delivered independently of the timing of radula movement cycles. Such behavioral findings therefore indicated that appropriate sensory stimuli can modify through learning processes the central network’s ability to “decide” when and how to act and convert the decision-making buccal circuitry into seemingly rigid and stereotyped rhythmogenic operation.

### Transformation of sporadic anticipatory burst activity into synchronized rhythmicity

**Figure 6:**
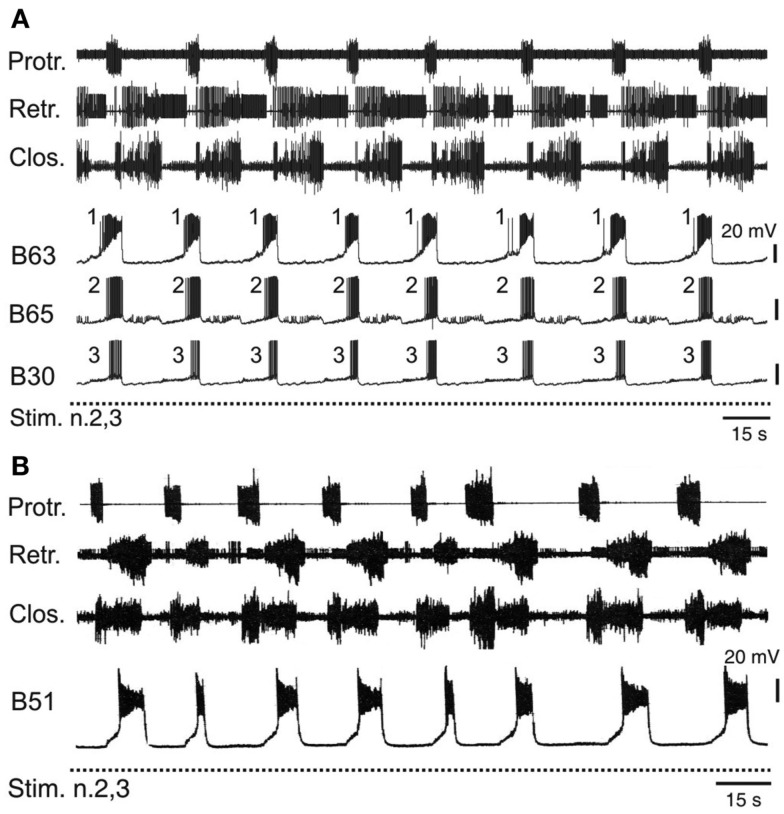
**Accelerated and stereotyped ingestive pattern genesis induced by appetitive operant learning**. **(A)** Simultaneous recordings of radula motor patterns and underlying bursting in the anticipatory B63, B65, B30 neurons in a buccal ganglia preparation isolated from a food reward trained animal. The rhythmic expression of motor patterns was associated with regular bursting in the anticipatory neurons, with each cycle being systematically led at a brief interval by burst onset in B63 (c.f. Figure 4B). **(B)** The regularization of ingestion pattern expression in an *in vitro* analog of appetitive learning was also associated with a systematic participation of B51 bursting in buccal CPG functioning. Compare with Figure 5A.

The operant learning-induced acquisition of rhythmic radula pattern generation was found to be associated with a synchronization of bursting activity in the anticipatory B63, B30, B65 neurons. In isolated buccal ganglia from operantly conditioned animals, not only were the delays between burst onsets in these neurons considerably decreased compared to their activity in ganglia from untrained animals, but also the order in which they became active in each pattern cycle became regularized (Figure 6A) such that bursts in the essential B63 neurons systematically commenced slightly before the burst onsets of the B65 and B30 neurons (Nargeot et al., 2009; Nargeot and Simmers, 2011).

The regularization of buccal network output was correlated to specific changes in the intrinsic membrane properties and the electrical coupling of the anticipatory protraction cells (Nargeot et al., 2009). In ganglia isolated from previously trained *Aplysia*, each of these neurons under conditions of functional isolation (as described above) spontaneously generated stereotyped, rhythmic bursts of action potentials, in contrast to the same cells in ganglia from untrained animals, which produced irregular and sporadic bursting. Concomitantly, the reduced variability in the process of motor pattern initiation was associated with a strong increase in the electrical coupling between the B63-B30 and B63-B65 cell pairs. The functional significance of this synaptic plasticity has been further investigated using the dynamic clamp technique (see Sharp et al., 1993) to artificially modify the strength of electrical coupling between these neurons (Nargeot et al., 2010; Sieling et al., 2010). In buccal ganglia from naïve preparations, which generate desynchronized anticipatory neuron activity and motor patterns with an irregular temporal distribution, an experimental increase in the electrical coupling between the B63-B30 and B63-B65 cell pairs regularized and synchronized their bursting and consequently, induced rhythmic motor pattern genesis. Conversely, in ganglia from trained animals, an artificial decrease in electrical coupling among the anticipatory neurons desynchronized their spontaneous burst onsets and switched their bursting and hence motor pattern genesis to erratic and irregular occurrences.

These recent findings therefore provide compelling evidence that cell-wide plasticity in the bioelectrical and synaptic properties of the anticipatory neuron subset serves as the causal link by which operant learning regulates the autonomous setting of occasions for the expression of radula feeding behavior. In a comparative context, it is also interesting that the synchronized neuronal and regularized network activity occurring in the buccal motor system after learning, now shares very similar features with other CPGs responsible for more conventional rhythmic behaviors. In the pyloric network of the crustacean stomatogastric nervous system, for example, rhythmogenesis arises from the endogenous oscillatory behavior of a tightly electrically coupled cell subset consisting of the AB and 2 PD pacemaker neurons (Selverston and Miller, 1980). The strongest oscillator AB entrains synchronized bursting in the 2PD cells and together they drive the rest of the pyloric circuit in a stereotyped triphasic pattern that produces rhythmic ingestive movements of the animal’s foregut (Selverston and Miller, 1980; Miller and Selverston, 1982; Bal et al., 1988).

### Stabilization of network configuration

In addition to modifying decisions about when to act, learning modifies the decision of how to act. After *Aplysia* makes the association of food reward with ingestive radula movements, the occurrence of this motor act increases at the expense of other actions in the animal’s behavioral repertoire (Nargeot et al., 2007). Correspondingly, in isolated buccal ganglia from such operantly conditioned animals, or in an *in vitro* analog of this associative learning, the buccal network generates the rewarded (ingestive) motor pattern to the detriment of the unrewarded egestion pattern (Figure 6; Nargeot et al., 1997, 2007; Brembs et al., 2002). In other words, learning modifies the process of motor pattern selection by “rigidifying” the buccal network into the specific functional configuration that ensures the continuous expression of the rewarded action.

Experimental evidence has indicated that this functional rigidity arises from a corresponding stabilization of the buccal circuit’s neuronal content. In ganglia from untrained animals, the B51 cells responsible for specifying ingestive pattern expression are only occasionally incorporated into network operation. In contrast, in previously trained preparations, B51 burst occurrences are strongly enhanced so as to systematically contribute, in a cycle-by-cycle manner, to motor pattern generation, and therefore to the predominance of ingestive pattern emissions (Figure 6B). Again, this reliable participation of B51 cells in buccal circuit functioning was attributable to learning-induced changes in their intrinsic membrane properties. In operantly trained preparations, the input resistance of these neurons and their probability of generating burst-producing plateau potentials were increased compared to B51 cells in untrained preparations (Nargeot et al., 1999a,b; Brembs et al., 2002; Mozzachiodi et al., 2008). Consequently, the depolarization of these cells via their electrical coupling with the retraction generator B64 neuron more reliably triggered bursting in time with each retraction phase, thereby resulting in the preponderance of ingestive pattern production.

Although a similar cellular analysis has not yet been extended to other neurons, such as B34 and B52 that are known to occasionally participate in buccal network operation and pattern selection processes, the above findings suggest that the reliable expression of a specific motor pattern phenotype is linked to a learning-derived specification and stabilization of the appropriate underlying circuitry. Here again, this rigidification in response to learning bestows the buccal CPG with functional features that are reminiscent of the relatively stable neuronal specification of the auto-active central networks responsible for typical rhythmic behaviors.

## Conclusion

The data summarized in this review indicate that the simpler and more accessible invertebrate CNS is endowed with neuronal correlates of elementary decision-making processes, including the selection and initiation of specific behavioral acts, which are amenable to cellular analysis. While sampling its environment in the search of food or during food consumption, *Aplysia* produces highly variable and sporadic movements of its buccal mass and rasp-like radula. This irregular goal-directed behavior is partly governed by the autonomous functioning of a central network that spontaneously selects and sets the occasions for the expression of distinct, sometimes opposing, radula actions.

**Figure 7:**
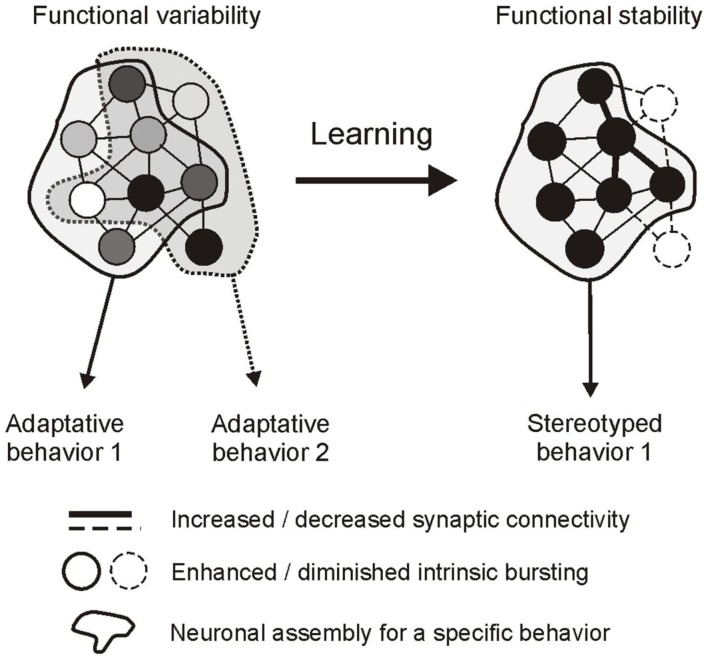
**Hypothetical representation of a decision-making network and its regulation by operant learning in *Aplysia***. Left, the global circuit is composed of a pool of neurons (circles) that generate erratic and weakly coordinated impulse bursts (indicated by different shading intensities) as a result of specific intrinsic properties and pattern of connectivity. The network can generate different adaptive behaviors depending on the participation of individual neurons. Some cells contribute to common features of the different behaviors (neurons in the overlapping area), others contribute selectively to a single behavior (neurons in the non-overlapping areas). Behavioral occasion setting is at least in part governed by the variability in burst coordination in the former subset of neurons, while behavioral selection depends on burst recruitment/exclusion in the latter subset. Right, learning rigidifies network functioning by modifying synaptic connectivity and intrinsic bursting properties. As a result, coordinated bursting now reduces the variability in occasion setting and pattern selection, allowing the expression of a single stereotyped rhythmic behavior.

Observations on still functioning buccal ganglia *in vitro* indicate that the auto-active network driving radula feeding movements shares fundamental properties with previously described CPG circuits responsible for automatic rhythmic behaviors. Major common features include: (1) a synaptic compartmentalization of the central network into distinct functional subcircuits that are each dedicated to a specific component of the CPG’s global output; and (2), a striking similarity in the dynamic membrane properties of the constituent neurons that underlie spontaneous network operation and the resulting patterned motor drive (see Marder and Calabrese, 1996; Selverston, 2010). However, several distinguishing features are also evident, particularly in relation to the capacity of a goal-directed CPG, such as *Aplysia’s* feeding network, to make internal decisions about the timing and nature of its behavioral expression. Thus, a salient feature of buccal network design is its functional complexity, involving a diversity of neuronal types within each functional subcircuit that are distinctive in terms of their individual patterns of synaptic connectivity, specific membrane properties, and therefore the spontaneity, timing and structure of their activity. A second distinguishing feature resulting from the first is the unpredictable variability of activity within and between the buccal CPG subsets. In the case of the protractor generator, irregular and weakly coordinated bursting amongst this unit’s anticipatory elements is able to govern the spontaneous setting of occasions for overall motor output expression. In an equivalent manner, the variability in burst expression and coordination between neurons that are essential to motor pattern genesis and non-essential circuit elements determines the selection between the different radula motor programs (Figure 7). On this basis, therefore, an elementary process in decision-making that enables the selection of goal-directed output and setting the occasion for its occurrence resides with the coordination of erratic spontaneous bursting within the functional subsets of the central network. It is also significant in this context that learning paradigms that regulate decision-making also regulate neuronal coordinating processes. As a result of sensory-mediated changes in synaptic connectivity and intrinsic membrane properties, variable cellular, and subcircuit activity is transformed into tightly coordinated and regular discharge (Figure 7), thereby converting otherwise unpredictable and erratic network output into a stereotyped rhythmic drive that now resembles the output commands of CPGs responsible for automatic behaviors.

Thus, in contrast to vertebrates where occasion setting and the selection of relevant goal-directed actions are thought to rely on distinct and functionally dedicated neural structures (see Balleine and O’Doherty, 2010), the buccal ganglia of *Aplysia* provide an intriguing example of how a single network, through autonomous multifunctioning mediated by specific synaptic and intrinsic neuronal properties, is able to achieve both tasks. Moreover, recent studies on this model system have offered a different conceptual framework for how reward learning can alter decision-making processes that may eventually lead to a habitual or compulsive expression of a particular goal-directed behavior. While data from vertebrates have suggested that learning switches the activation of decision-making circuitry to the recruitment of distinct automatic networks for habitual behavior (see Balleine and O’Doherty, 2010), recent findings in *Aplysia* indicate that the conversion of a goal-directed act to an automatic and rhythmic behavior can arise from a learning-induced rigidification in the functional properties of the decision network itself. A greater understanding of the cellular and sub-cellular mechanisms underlying decision-making in goal-directed behaviors of invertebrates may therefore provide general insights into the neuronal basis of decision-making processes and their regulation by learning or their deregulation in behavioral disorders.

## Conflict of Interest Statement

The authors declare that the research was conducted in the absence of any commercial or financial relationships that could be construed as a potential conflict of interest.
